# Experimental Investigation of the Acoustic Nonlinear Behavior in Granular Polymer Bonded Explosives with Progressive Fatigue Damage

**DOI:** 10.3390/ma10060660

**Published:** 2017-06-16

**Authors:** Zhanfeng Yang, Yong Tian, Weibin Li, Haiqiang Zhou, Weibin Zhang, Jingming Li

**Affiliations:** 1 Institute of Chemical Materials, China Academy of Engineering Physics, Mianyang 621900, China; yangzf@caep.cn (Z.Y.); haiqiang0602@caep.cn (H.Z.); weibinzhang1@163.com (W.Z.); jmli7288@caep.cn (J.L.); 2 School of Aerospace Engineering, Xiamen University, Xiamen 361005, China

**Keywords:** polymer bonded explosives, micro-cracks, acoustic nonlinearity, fatigue

## Abstract

The measurement of acoustic nonlinear response is known as a promising technique to characterize material micro-damages. In this paper, nonlinear ultrasonic approach is used to characterize the evolution of fatigue induced micro-cracks in polymer bonded explosives. The variations of acoustic nonlinearity with respect to fatigue cycles in the specimens are obtained in this investigation. The present results show a significant increase of acoustic nonlinearity with respect to fatigue cycles. The experimental observation of the correlation between the acoustic nonlinearity and fatigue cycles in carbon/epoxy laminates, verifies that an acoustic nonlinear response can be used to evaluate the progressive fatigue damage in the granular polymer bonded explosives. The sensitivity comparison of nonlinear and linear parameters of ultrasonic waves in the specimens shows that nonlinear acoustic parameters are more promising indicators to fatigue induced micro-damage than linear ones. The feasibility study of the micro-damage assessment of polymer bonded explosives by nonlinear ultrasonic technique in this work can be applied to damage identification, material degradation monitoring, and lifetime prediction of the explosive parts.

## 1. Introduction

Polymer bonded explosives (PBX) which are particle filled composite materials comprised of powerful secondary explosive crystals and held together by a polymeric binder, are widely used in both military and civil applications [1,2]. As an energetic material, PBX may be subjected to various external stimuli—such as compression, tension, or impact—which will cause micro-structural change, and even the initiation and progression of micro-damage [3]. The micro-damage accumulated via fabrication, storage, and in-service processing of PBXS can significantly degrade the mechanical properties, and greatly affects their combustion and detonation ability [4]. Considering the unique characteristics and importance of safety of PBX material, nondestructive assessment of micro-damage at early stages is essential for the prevention of unpredictable catastrophic failures initiated from micro-damage to PBX.

Optical and electronic microscopic examinations have been used to evaluate the damage and microstructures of PBX [5]. These methods can observe the initial spatial damage distribution of the whole specimen. However, they are destructive, time consuming, and often unable to assess the samples in loading process. Recently, damage evolution in PBX was characterized by using acoustic emission and digital image correlation to evaluate the spatial distribution of damages [6]. Earlier studies also investigated the damage and fracture characteristics of PBXs based on technology of acoustic emission [7,8,9]. Compared to optical and electronic microscopic methods, acoustic emission is a nondestructive method and it can be used to track the damage progression process in solids. However, acoustic emission is a passive test approach, in that it is based on the detection of wave signals emitted by growth and deformation of defect or failure in the specimen. It is difficult to monitor the defects with constant size or material aging degradation by this method.

Ultrasonic nondestructive evaluation (NDE) is well known for its application to quantify the basic mechanical and structural properties of solids and it can be used for flaw detection, dimensional measurements, and material characterization. However, it is understood that the presence of microscopic imperfections is produced by early micro-damage and subsequently serve as nuclei of the fracture process, which can significantly affect the material properties. Conventional linear ultrasonic techniques are usually less sensitive to this kind of microscopic degradation of the materials, since the acoustic wavelength used in ultrasonic NDE is generally not as short as the size of these nuclei. Considering the high sensitivity of nonlinear ultrasonic technique for micro-damage and micro-structural evolution in solid media, the use of acoustic nonlinear response has drawn significant attention for nondestructive evaluation of micro-damage in early stage [9,10,11,12]. The typical nonlinear phenomenon is the generation of second harmonics, i.e., the formation of a harmonic frequency that is double the frequency of the fundamental input frequency, which results from waveform distortion in the time domain. The use of second harmonic generation has been reported in numerous studies to assess the micro-damage of materials [13,14,15,16] and characterize material degradation [17,18]. It has been shown that nonlinear ultrasonic waves can be used for the evaluation of material nonlinearity and hence are taken as the sensitive indicators for early stage damage.

In this paper, acoustic nonlinear response of ultrasonic wave propagation in PBXs is investigated to assess the fatigue induced micro-cracks in earlier stage. The variation of second harmonic amplitude is used to characterize the evolution of fatigue induced micro-cracks. The sensitivity comparison of linear and nonlinear ultrasonic parameters to micro-damage is also discussed in this work. Based on the results, a promising experimental procedure is developed to indicate micro-damage at early stages in polymer bonded explosive components using nonlinear ultrasonic technique.

## 2. Experiments

PBXs are a kind of granular material, the micro-cracks are the main source for the acoustic nonlinearity for the material fatigue [19,20,21,22]. The physical nature of the micro-crack induced acoustic nonlinearity can be explained by the consideration of ultrasonic wave propagation through a contact between two solid surfaces. The discontinuities of stress and displacement will cause the acoustic nonlinearity. Let a longitudinal ultrasonic wave propagate through a contact between two rough surfaces, the initial contact area increases in the compression phase, while it decreases in the rarefaction phase [23,24,25,26]. As a result, the amplitudes of the transmitted and reflected acoustic waves also vary in accordance with variations in the area of contact surfaces. Therefore, the transmitted and reflected waves acquire amplitude modulation with a frequency of the external effect which is different from the incident waves [27].

The aim of detecting material nonlinearity induced by micro-defects is to measure the acoustic nonlinearity, which can be represented by the second harmonic amplitude over the square of that of fundamental one. The formation of a harmonic frequency that is double the frequency of the fundamental input one, results from the waveform distortion in the time domain after a certain propagation distance. The ultrasonic wave is distorted due to material nonlinearity, and consequently, higher harmonics are generated. Thus, the received signal is composed of not only the fundamental frequency wave but also second or higher harmonics frequency wave. In this work, the measurement of micro-damage is typically aimed at determining the value of nonlinear acoustic parameter *β*. The nonlinear parameter is related to the amplitudes of the fundamental wave and second harmonics [21,22]

*β*’=*A*_2_/*A*_1_^2^ ∝*β* (1)

where *A*_1_ and *A*_2_ are the amplitude of fundamental wave and the second harmonic wave, respectively. The ratio can be tested experimentally. Thus, the material nonlinearity can be evaluated by detecting the fundamental and the second harmonic amplitudes. For the qualitative evaluation of material nonlinearity, all of the values are normalized to represent only the relative change of acoustic nonlinear response.

The specimens used in this study are TATB-PBXs with cylinder shape of size *Φ* 20 × 20 mm. There were various kinds of damages in PBXs materials, such as granulation crushing, matrix cracking, micro-fissure and interface debonding et al., which could degrade mechanical properties. The most common approach to create artificial damage in PBXs is by controllable tension. In order to introduce micro-damage into the specimen, the uniaxial compression fatigue loadings with constant pressure were conducted on the TATB-based PBX specimen. The tested specimens were subjected to an interrupted mechanical test with 200, 250, 300, and 350 cycles of fatigue loading with a MTS-858 testing machine (Eden Prairie, MN, USA), then, removing the mechanical loading to make a set of ultrasonic measurement. For one mechanical fatigue cycle, the specimen is compressed in the test machine under 5.625 KN for 10 minutes, and then is unloaded for another 10 min.

**Figure 1:**
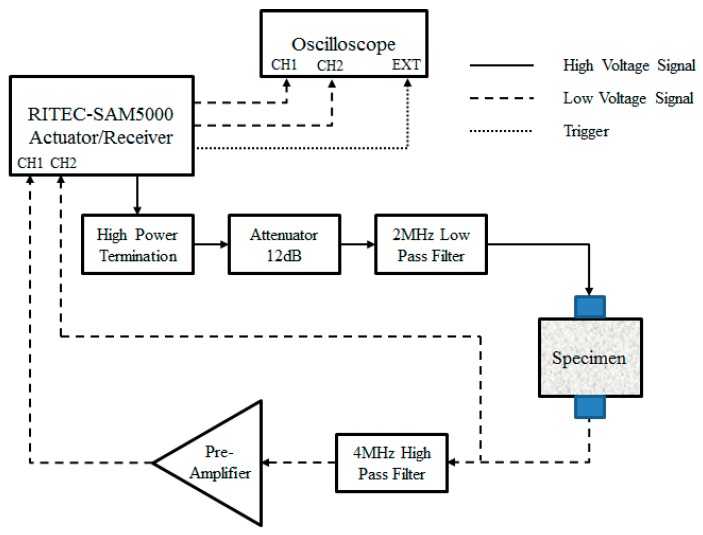
Block diagram of ultrasonic measurement system.

The experimental setup for measuring the acoustic nonlinear response is shown in Figure 1. In the experimental setup, a high-power termination is connected to the actuator to generate a high-power narrow band signal with a central frequency of 2 MHz. The signal transmitted through the 12 dB attenuator and the 2 MHz low pass filter couplant to minimize the effect of instrumentation on incident signal. The low pass filter is used to eliminate the higher harmonic frequency components induced by instruments or couplant. The attenuator is set to purify the signal for a high ratio of signal-to-noise. A 4 MHz high pass filter and pre-amplifier are used to primary measure the double frequency second harmonic component. The transducers are coupled to the specimen with light lubrication oil. Both transducers are carefully placed on each side of the specimens with holders designed to ensure uniform coupling conditions.

**Figure 2:**
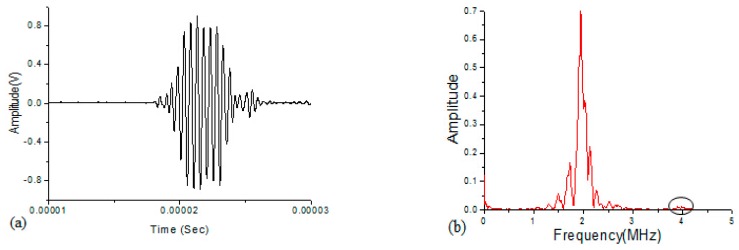
Experimental time domain signal (**a**) and (**b**) frequency spectrum (FFT) of the signal.

In this study, averaging is applied to time-domain waveforms prior to computing the FFT to reduce noise. Time domain signals were recorded and averaged 256 times with an oscilloscope, and then transferred to a computer for further signal processing. A Hanning window is imposed on the steady state part of the signal and signals are digitally processed by using the Fast Fourier Transformation (FFT) to obtain amplitudes *A*_1_ at the fundamental frequency and *A*_2_ at the double frequency, respectively. As shown in Figure 2, a typical ultrasonic signal obtained in experiments is provided as an example. Meanwhile, linear ultrasonic tests based on measurement of acoustic velocity are also carried out for the comparison of sensitivity for material characterization by linear and nonlinear acoustic parameters.

## 3. Results and Discussion

### 3.1. Verification of the Proposed Method

**Figure 3:**
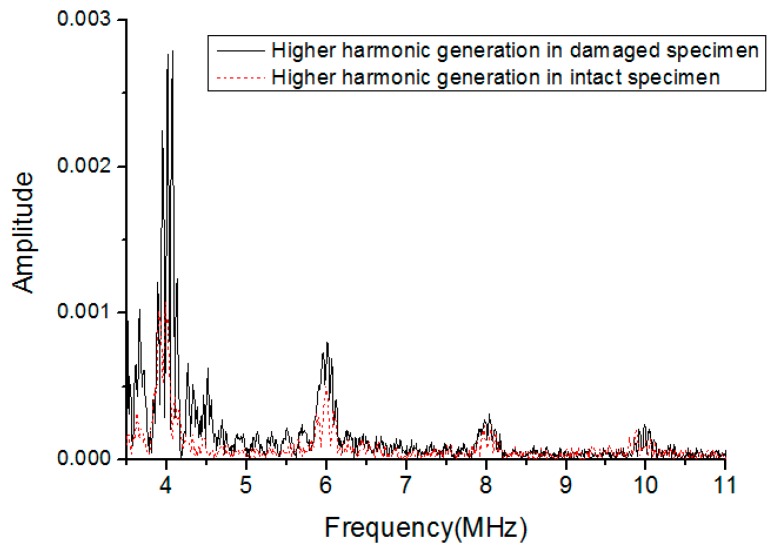
Comparison of higher harmonic generation in intact and damaged specimens.

The propagation of ultrasonic waves through the PBX materials with debonding, micro-cracks, uncoating, et al., will cause the change of magnitude of the ‘contact’ pressure, thus a local opening and closure of the interface will be generated by the ultrasonic waves. The variations of this contact condition will also distort the propagating waveform [28,29]. As discussed above, the physical effect of acoustic nonlinearity is nothing more than generation of higher harmonics which caused by the waveform distortion. As the damage states increase in the specimen, the distortion of waveform will also be changed. Without a doubt, there could be many other nonlinear sources coming from experimental set-up besides material micro-damage. To assure that the nonlinearity from material damage is dominated over other sources. Only the nonlinear response noticeable exceeding the base ling value of the intact specimens is used for the analysis, as shown in Figure 3.

**Figure 4:**
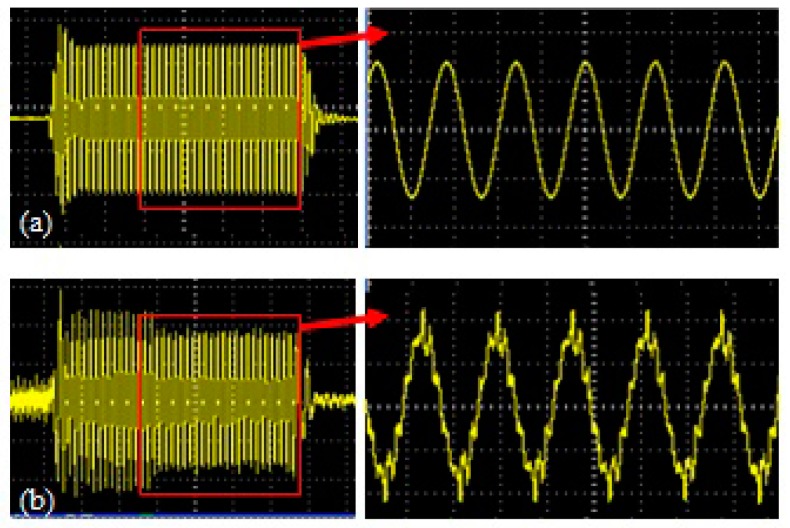
Experimental waveforms of ultrasonic waves in the specimens with higher density (**a**) and (**b**) lower density.

To verify reliability of the proposed approach for the characterization of micro-damage in the specimens, acoustic nonlinear response of ultrasonic waves in the specimens with different density were measured. PBXs samples are generally formed by pressured molding, thus, the lower density of specimen corresponds to the higher inattentive micro-structures, and more micro-damage in the specimens [30,31,32,33,34,35]. It is shown in the Figure 4 that the waveform of ultrasonic signals in the specimen with lower density is much more distorted, which means the acoustic nonlinear response is much stronger.

**Figure 5:**
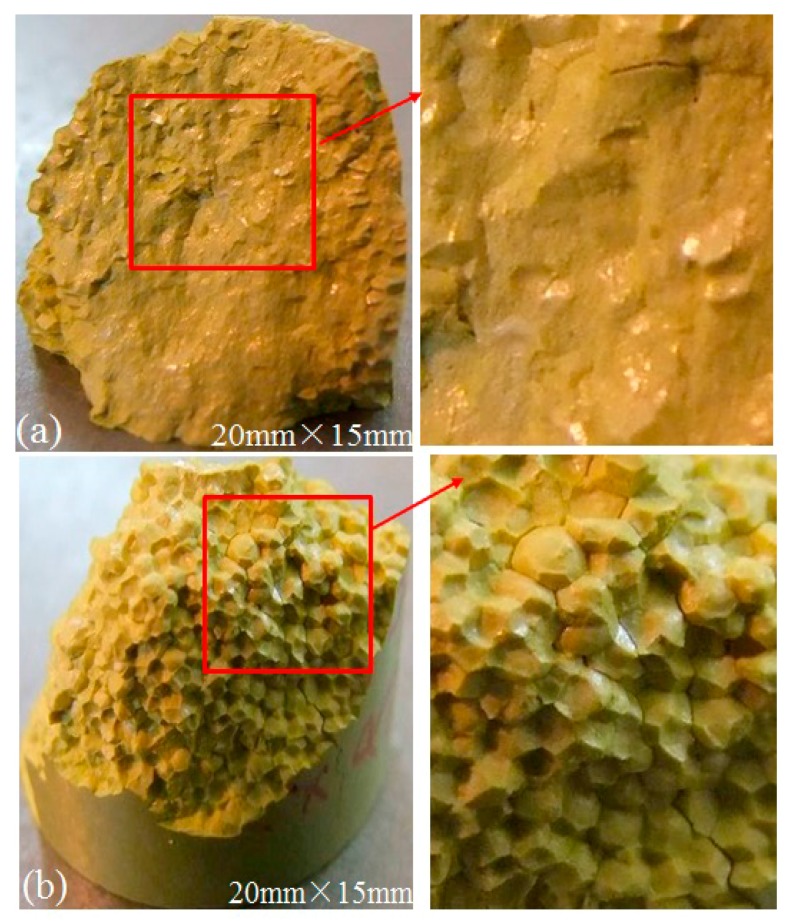
Micrographs of specimens with (**a**) higher density and (**b**) lower density.

To supplement the features observed in the nonlinear ultrasonic testing, microstructural changes of the specimens with different densities are also observed by optical microscope, as illustrated in Figure 5. It shows that the fissure and cracks observed in the specimen with higher density are much less than those in the one with lower density. It can be concluded that, the granular coated quality of specimen with lower density is worse than that with higher density, as well as the bonding strength between granular interfaces. The results displayed in the figure agree well with the prediction from the nonlinear ultrasonic tests. The compression breaking experiment and fracture analysis showed that the initial damage and damage degree could be determined qualitatively and nondestructively by the nonlinear ultrasonic coefficient.

### 3.2. Characterization of the Progressive Fatigue Micro-Damages by Acoustic Nonlinearity

**Figure 6:**
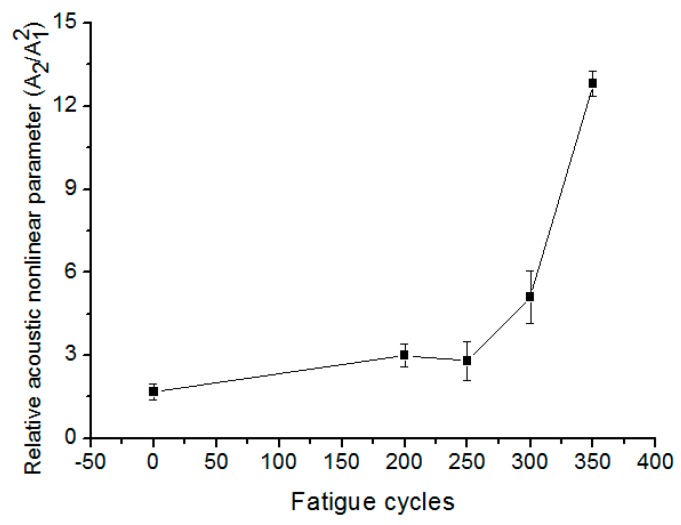
Variation of relative acoustic nonlinear parameter versus fatigue cycles.

Fatigue cyclic loading induced micro-damage is the strongest source of nonlinearity. As the increase of fatigue cycles, the variations of acoustic nonlinear parameters will be used to quantitatively characterize the evolution of micro-damage. The fatigue tests are interrupted to perform the nonlinear ultrasonic measurements at different numbers of fatigue cycles. Figure 6 shows the correlation of the fatigue cycles and the relative nonlinear parameters. As the fatigue cycles increase, the values of acoustic nonlinearity change significantly. Error bars of the measured result have been added.

**Figure 7:**
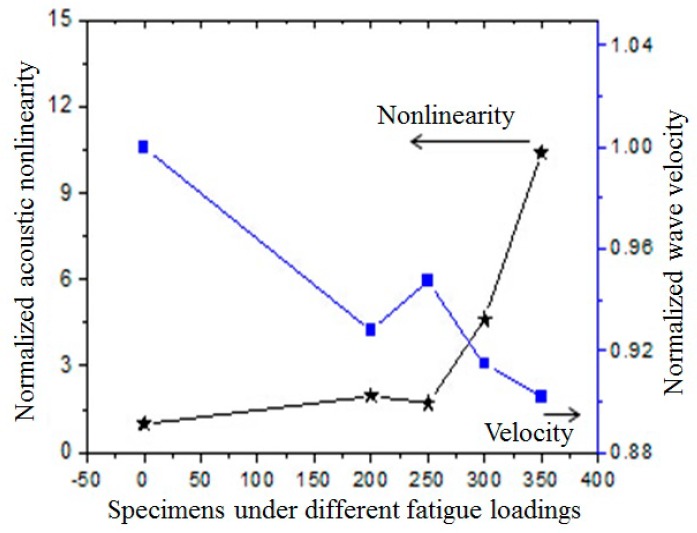
Comparison of variations of acoustic nonlinear parameter and wave velocity to fatigue loadings in the specimens; the acoustic nonlinearity and wave velocity are normalized by the reference values of the raw material, ${\bar{\beta }}_{Raw}$ = 1.31 and C_Raw_ = 2.323 km/s, respectively.

Linear ultrasonic test based on the measure of wave velocity is also carried out to compare with the nonlinear ultrasonic approach. To display only the relative change, all measured acoustic parameters are normalized by the values in the intact specimen. As shown in Figure 7, the typical evolution of the normalized acoustic nonlinearity and wave velocity are plotted with the change of fatigue cycles. Observations of relative acoustic nonlinearity shows two peaks after 250 cycles and before 300 cycles. Almost the same trend of velocity variation is also observed. At the initial stage, as the increase of fatigue cycles, the crack nucleation, and growth are the main sources of nonlinearity. The nonlinearity should increase monotonically as the number of small cracks increases. The drop of nonlinearity after 250 cycles can be interpreted as the reason of interactions of the crack, the presences of cracks will release the residual stress, micro-debonding, and other micro-damages. Thus, the density of micro-damages will decrease. However, as the fatigue cycles increase, the significant increase of nonlinearity will show up, which means that once the cracks are initiated, they can grow relatively quickly and lead to catastrophic failure. The nonlinearity parameter increases more or less exponentially, with a local decrease at 250 loading cycles. The velocity change can be interpreted in the same way, although it is less sensitive to fatigue induced micro-damage.

**Figure 8:**
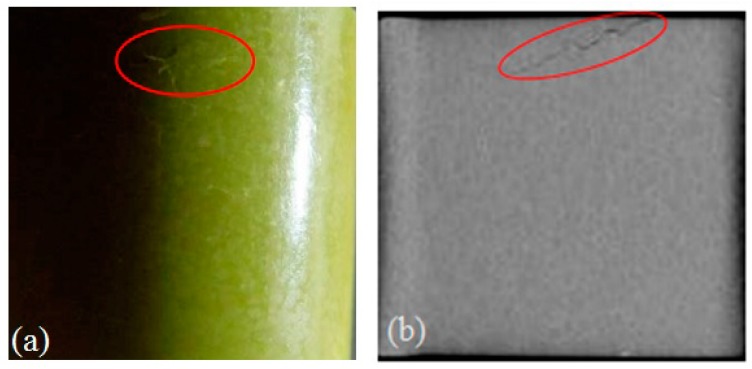
Optical (**a**) and computer tomographical image (**b**) of the specimen after 350 fatigue cycles.

To supplement the features observed in the ultrasonic testing, pictures of surface crack and crack initiation inside the specimen are also obtained by computer tomographical scanning as illustrated in Figure 8. The results displayed in the figure agree well with the prediction from the acoustic nonlinearity test. It shows the dramatic progressive fatigue damage after 350 cyclic loading. The value of acoustic nonlinearity also increases significantly, which is almost 10 times bigger than the initial one. As a result, overall the data trend matches well with the observed results of acoustic nonlinearity. The result demonstrates that nonlinear ultrasonic wave approach is promising for characterizing the evolution of micro-damage in PBX material. Note that the technique is investigated as a qualitative monitoring tool, not a quantitative one, in terms of the correlation between damage level and ultrasonic nonlinearity.

## 4. Conclusions

The use of acoustic nonlinearity is proposed to evaluate and detect the micro-damage in TATB based PBX material in this investigation. Variation of second harmonics of ultrasonic waves propagation in the specimens are measured and taken as the indicators of evolution of micro-damages. The proposed approach was firstly conducted in the two specimens with different initial micro-damage density to verify the reliability of the technique. The sensitivity comparison of nonlinear and linear parameters of ultrasonic waves in the specimens shows that nonlinear acoustic parameters are more promising indicators of fatigue damage than linear ones. The feasibility study of the detection of the micro-damage of polymer bonded explosive components by nonlinear ultrasonic technique in this work, can be applied to damage identification, material degradation monitoring, and lifetime prediction of explosive parts.
